# Size doesn't matter, sex does: a test for boldness in sister species of *Brachyrhaphis* fishes

**DOI:** 10.1002/ece3.1304

**Published:** 2014-10-27

**Authors:** Spencer J Ingley, Jeremy Rehm, Jerald B Johnson

**Affiliations:** 1 Evolutionary Ecology Laboratories, Department of Biology, Brigham Young UniversityProvo, Utah, 84602; 2 Monte L. Bean Life Science Museum, Brigham Young UniversityProvo, Utah, 84602

**Keywords:** Behavioral divergence, boldness, *Brachyrhaphis*, life-history trade-offs, predation

## Abstract

The effect of divergent natural selection on the evolution of behavioral traits has long been a focus of behavioral ecologists. Predation, due to its ubiquity in nature and strength as a selective agent, has been considered an important environmental driver of behavior. Predation is often confounded with other environmental factors that could also play a role in behavioral evolution. For example, environments that contain predators are often more ecologically complex and “risky” (i.e., exposed and dangerous). Previous work shows that individuals from risky environments are often more bold, active, and explorative than those from low-risk environments. To date, most comparative studies of environmentally driven behavioral divergence are limited to comparisons among populations within species that occur in divergent selective environments but neglect comparisons between species following speciation. This limits our understanding of how behavior evolves post-speciation. The Central American live-bearing fish genus *Brachyrhaphis* provides an ideal system for examining the relationship between selective environments and behavior, within and between species. Here, we test for differences in boldness between sister species *B. roseni* and *B. terrabensis* that occur in streams with and without piscivorous predators, respectively. We found that species do differ in boldness, with species that occur with predators being bolder than those that do not. Within each species, we found that sexes differed in boldness, with males being bolder than females. We also tested for a relationship between size (a surrogate for metabolic rate) and boldness, but found no size effects. Therefore, sex, not size, affects boldness. These results are consistent with the hypothesis that complex and risky environments favor individuals with more bold behavioral traits, but they are not consistent with the hypothesis that size (and therefore metabolic rate) drives divergence in boldness. Finally, our results provide evidence that behavioral trait divergence continues even after speciation is complete.

## Introduction

The effect of divergent selective environments on the expression and evolution of divergent behavioral traits has long been a focus of behavioral ecologists (Foster 1999; Foster and Endler 1999). Numerous environmental factors can act as selective agents on behavioral traits. For example, behavioral differences among populations or species can result from both biotic (e.g., community dynamics) and abiotic (e.g., habitat complexity) factors. Furthermore, divergence in behavioral traits among species or populations from different environments can represent genetic divergence, phenotypic plasticity, or both (West-Eberhard 2003; Foster 2013). Behavioral ecologists have long turned to comparative studies of species or populations that occur in different selective environments as a way to identify the relative contribution of biotic and abiotic mechanisms to the variation in behavior.

Predation is among the most well studied ecological drivers of phenotypic divergence among populations or species from different environments (Endler 1987; Huntingford et al. 1994a; Reznick 1996; Johnson 2001; Johnson and Belk 2001). Predation affects a variety of prey traits, including morphology (Langerhans and Dewitt 2004; Ingley et al. 2014a), life history (Reznick and Bryga 1987; Johnson 2001; Johnson 2002; Johnson and Zuniga-Vega 2009), and behavior (Huntingford et al. 1994; Godin and Briggs 1996; Brown and Braithwaite 2005). Recently, the impact of predation on behavior has received considerable attention (Ioannou et al. 2008; Harris et al. 2010; Archard and Braithwaite 2011a; Archard et al. 2012). In addition to direct effects of predation on traits such as behavior, the presence of predators is also often confounded with a variety of environmental factors that can be highly correlated with the presence or absence of predators (Johnson 2002; Ingley et al. 2014a). For example, habitats with or without predators often differ in terms of fish and invertebrate community complexity, available microhabitats, and prey types (Archard and Braithwaite 2011a). The behavioral response of populations from different “predation environments” (i.e., those that are predator-naïve and occur in less complex habitats versus those that are predator-exposed and occur in more complex habitats) remains a question of great interest in behavioral ecology (Archard and Braithwaite 2011a).

One behavior that has been studied in the context of divergent environmental differences, including predation, is the propensity to take risks, or “boldness” (Brown and Braithwaite 2004; Urban 2007; Adriaenssens and Johnsson 2011). Boldness has been a focal behavioral trait because of the impact that an individual's boldness can have on numerous processes, such as reproductive and life-history strategies (Witsenburg et al. 2010), exploration tendencies (Wilson and Godin 2009; Rodriguez-Prieto et al. 2011), resource use and acquisition (Adriaenssens and Johnsson 2011), or mate selection (Godin and Dugatkin 1996). For example, researchers have found that boldness plays an important role in dispersal tendencies (Fraser et al. 2001; Dingemanse et al. 2003), mate choice (Godin and Dugatikin 1996), aggression toward conspecifics (Archard and Braithwaite 2011b), and survival in the presence of a predator (Magurran and Seghers 1990). Although findings have been mixed among a diversity of taxa, individuals from populations that occur in predator-rich environments are often more bold than individuals that occur in predator-free environments (Urban 2007; Archard and Braithwaite 2011a). However, the direction of selection on boldness traits (i.e., if selection favors increased or decreased boldness levels) is likely highly context specific, particularly because both predation and other environmental factors can affect boldness between populations of a given species (Brydges et al. 2008). For example, on one hand, increased boldness levels in prey could increase their risk of predation if bold individuals are also more active, and prey movement facilitates detection by predators [e.g., where predators actively pursue prey; (Kruuk and Gilchrist 1997). On the other hand, if a bold individual spends more time in “exposed” microhabitats (i.e., away from predator habitat, in the case of sit-and-wait ambush predators), it may be able to better avoid predators by identifying and avoiding the predator's location. In either case, boldness is likely to play an important role in an individual's interaction with the environment, conspecifics, and heterospecifics, thus underscoring the importance of this behavioral trait.

Divergence in boldness traits among or within populations or species from divergent environments could also be explained by trade-offs in life-history strategies (Wolf et al. 2007). For example, despite the potential costs associated with risky behaviors (e.g., increased predation risk), bold behavior could pay off if bold individuals increase mating opportunities and maximize current reproduction due to increased encounter rates with potential mates (Wolf et al. 2007). Increasing encounter rates with potential mates could be particularly important in predator environments that are often characterized by low population densities (i.e., fewer encounter opportunities with potential mates; Abrams 1993) and a higher risk of predator-induced mortality. Males and females within species are also likely to differ in boldness due to differences in reproductive strategies (Harris et al. 2010). For example, males suffer a lower cost of mating than females, potentially favoring more active and bold behavioral phenotypes in males in order to maximize mating opportunities. Furthermore, more bold individuals, which are often more prone to explore novel environments, could be better at locating and exploiting the more complex array of microhabitats typical of predator-rich environments. It is thus reasonable to hypothesize that populations that occur in predator-rich environments will be bolder than their predator-naïve congeners.

Aquatic systems are well suited for studying the processes that drive behavioral divergence (Riechert 1999), particularly with traits related to predation. This is due in part to the relative ease with which abiotic and biotic factors can be characterized in aquatic systems (e.g., Johnson 2002), and the fact that populations within a species can often be found in numerous water bodies representing a diversity of predation environments (e.g., from absence to abundance of predators). Fish have thus become a major focal group for studying behavior, including boldness, in predatory contexts. Among the most well-studied fish groups are members of the live-bearing fish family Poeciliidae (Johnson 2001; Jennions and Kelly 2002; Basolo 2004; Brown and Braithwaite 2005; Mateos 2005; Jones and Johnson 2009; Wesner et al. 2011). Numerous studies on live-bearing fishes have focused on the impact of predation on several traits, including morphology, life history, and behavior (Reznick and Bryga 1987; Reznick 1989; Rodd and Reznick 1991; Johnson and Belk 2001; Reznick et al. 2001; Brown and Braithwaite 2004; Langerhans 2009a,b; Langerhans and Makowicz 2009; Wesner et al. 2011). Several species within the genus *Brachyrhaphis* have provided excellent examples of how predation shapes life history (Johnson and Belk 2001), morphology (Ingley et al. 2014a) and behavior (Archard and Braithwaite 2011a,b; Ingley et al. 2014b). *Brachyrhaphis* occurs primarily in lower central America, with most species indigenous to Costa Rica and Panama. Numerous species of *Brachyrhaphis* exhibit divergence in several traits corresponding to differences in predation environment and show signs of population divergence and speciation driven by differences in predation environment. Populations of *Brachyrhaphis rhabdophora* and *B. episcopi,* for example, show different life-history strategies (Johnson and Belk 2001; Jennions and Telford 2002), body shapes (Ingley et al., 2014), and behavior (Brown and Braithwaite 2005; Archard and Braithwaite 2011a; Ingley et al. 2014b) associated with predation environment that are similar to those observed in other poeciliid species (Reznick 1996; Langerhans and Makowicz 2009; Harris et al. 2010). Although past efforts have focused primarily on predator-driven divergence among populations of a single species (e.g., within *B. rhabdophora* or *B. episcopi*), recent work suggests that similar patterns could also exist at the between species level (e.g., Ingley et al. 2014a). Our study takes advantage of the unique natural history of sister taxa of poeciliid fishes *Brachyrhaphis roseni* and *B. terrabensis* that exhibit similar patterns of divergence to those observed in previous studies of fish from different predation environments (Ingley et al. 2014a), but between sister species rather than among populations within a single species.

*Brachyrhaphis roseni* and *B. terrabensis* have similar distributions, occurring from southeastern Costa Rica to central Panama (Bussing 1998), and typically occupy the same stream systems. Even so, *B. terrabensis* lives in higher elevation headwater streams, whereas *B. roseni* occupies lower elevation coastal streams (Bussing 1998). Consequently, *B. terrabensis* occurs in environments primarily free of piscivorous predators, whereas *B. roseni* lives in complex lowland stream environments with a variety of predatory fish (e.g., *Hoplias microlepis* and *Gobiomorus dormitor*). Both species have evolved similarly divergent life histories (M. Belk et al., in review) as those observed among populations of *B. rhabdophora* (Johnson and Belk 2001) and *B. episcopi* (Jennions and Telford 2002). Body shape also varies within *B. rhabdophora* (Langerhans and Dewitt 2004) and between *B. roseni* and *B. terrabensis* (Ingley et al. 2014a), in each case as predicted by locomotor trade-offs associated with different predation environments. The fact that *B. roseni* and *B. terrabensis* are sister taxa and that they occur in close geographic proximity, but in divergent predation environments suggests that predation might be the primary driver of speciation in this group. This makes *B. roseni* and *B. terrabensis* an ideal system to examine how boldness evolves in response to different environments, even after speciation is complete. Thus, we examine how boldness evolves in response to different environments once speciation is complete by addressing the following objectives. First, we examined the boldness levels of *B. roseni* and *B. terrabensis* to determine if divergence in behavioral traits was present. In accordance with previous studies and our own hypotheses outlined above, we expected that *B. roseni,* which lives with predators, would be bolder than *B. terrabensis*, which does not. Second, we tested for differences in boldness traits between the sexes in each of these species to see if males and females differed, with the prediction that males would be bolder than females due to differences in life-history strategies. Lastly, we tested for significant associations between size and boldness across our entire data set, both within species and within sexes of each species, as size (a surrogate for metabolic rate) has been implicated as a driver of behavioral divergence in similar studies (Brown and Braithwaite 2004).

## Methods

### Live fish collection and care

In February of 2013, we used handheld seines to collect live *Brachyrhaphis roseni* and *B. terrabensis* from two streams (one site/population per species) in the Rio Chiriquí Nuevo drainage in Chiriquí, Panama. We collected *Brachyrhaphis roseni* from a small, low-elevation tributary (N 8.424914, W 82.416065, elevation: 18 meters), and *B. terrabensis* from a small, high-elevation tributary (N 8.76577, W 82.43379, elevation: 1073 meters). We immediately transported fish to 300-liter community pools where they were held for at least 7 days prior to testing (at densities of approximately 80 fish per pool). We fed fish TetraColor Tropical Flakes twice daily while in the holding pools and kept them outdoors under natural light (∼12L: 12D) and temperature conditions (24–26°C).

### Experimental assay for boldness

We conducted controlled behavioral trials to measure time to emerge from a shelter into a novel environment. This metric is commonly used in behavioral studies [e.g., (Brown et al. 2005, 2007; Harris et al. 2010)] and assumes that fish that emerge into a novel environment more quickly are bolder than fish that emerge into a novel environment more slowly (Toms et al. 2010). We conducted our test for boldness in an experimental arena that consisted of a 63.5 cm × 41.5 cm × 26 cm tank attached to separate acclimation chamber measuring 31 cm × 20.5 cm × 26 cm. The acclimation chamber was connected to the test arena by a 14.5 cm × 16 cm opaque cylinder. We installed an opaque plastic door to prevent the fish from exiting the acclimation chamber prior to the start of the trial. This door could be removed remotely via pulley. For all trials, water was filled to 13 cm.

Two days prior to testing, we haphazardly selected approximately equal numbers of males and females (only adults were used in our study) for each species from the communal pools and placed them in individual 5-liter holding tanks. For each trial, we carefully removed a focal fish from the holding tank and placed it in the acclimation chamber, where it remained for a 2-minute acclimation period. Following the acclimation period, we removed the trap door via pulley to allow the subject to freely move between the acclimation chamber and the novel tank environment. We video recorded each trail using a LogitechC260 webcam that was mounted above the test arena. For each trial, we blindly scored the latency (i.e., duration of time from the start of the trial) to emerge into the novel environment from the acclimation chamber. We considered a fish to have emerged when its entire body had crossed the threshold between the acclimation chamber and the novel tank environment. Each trial was terminated at 10 minutes. For fish that did not exit the chamber prior to the end of the 10-min period, we assigned a maximum score of 600 sec. At the conclusion of each trial, we removed the subject from the test arena, anesthetized it using MS-222, and took standard length (SL) and mass measurements. In total, we tested 38 male and 37 female *B. roseni,* and 34 male and 25 female *B. terrabensis*. All collecting and experiments were reviewed and approved by the Smithsonian Tropical Research Institute and Brigham Young University IACUC committees.

### Statistical analysis

To determine if species and sexes differed in their time to emerge, we conducted an analysis of covariance (ANCOVA) with species and sex as main factors, SL as a covariate, and time to emerge as the response variable. Two- and three-way interaction terms between sex, species, and SL were also included. For each term, we calculated effect size as partial *η*^*2*^. To meet the assumptions of ANCOVA, we natural log transformed our time to emerge data. This ANCOVA allowed us to control for any effect of size on boldness, as *B. roseni* and *B. terrabensis* significantly differ in both SL and mass (see Results). We also used ANCOVA to test for differences in time to emerge between sexes within each species, with sex as a factor, SL as a covariate, and the log of time to emerge as the response variable. Finally, we used ANOVA to test for differences in SL between species and sexes and to compare time to emerge between sexes within species. To compare our results to previous work (Brown and Braithwaite 2004), we used linear regression to test for a relationship between time to emerge (i.e., boldness) and SL for the entire data set (mass and SL were highly correlated [*F*_1, 132_ = 1087, *P <* 0.001, adjusted *R*^2^ = 0.89], so we chose to focus on SL alone in our linear regression analyses). We then conducted linear regression analyses for each species to determine if there was a relationship between time to emerge and SL. Finally, we conducted linear regression analyses for each sex by species combination to determine if, within each sex for each species, there was a relationship between time to emerge and SL. All analyses were conducted in R (R-core development team, 2008).

## Results

**Figure 1:**
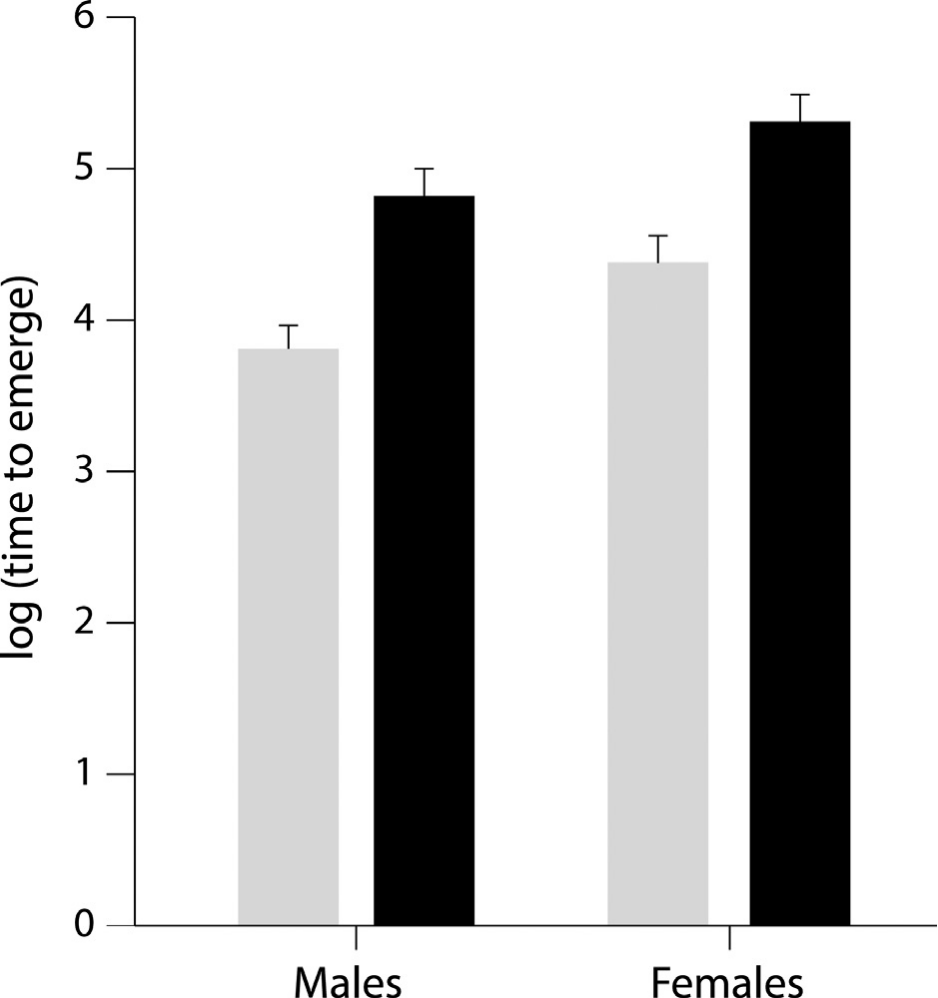
Means ± standard error (SE) of the log of time to emerge for males and females of *Brachyrhaphis roseni* (gray) and *Brachyrhaphis terrabensis* (black).

**Figure 2:**
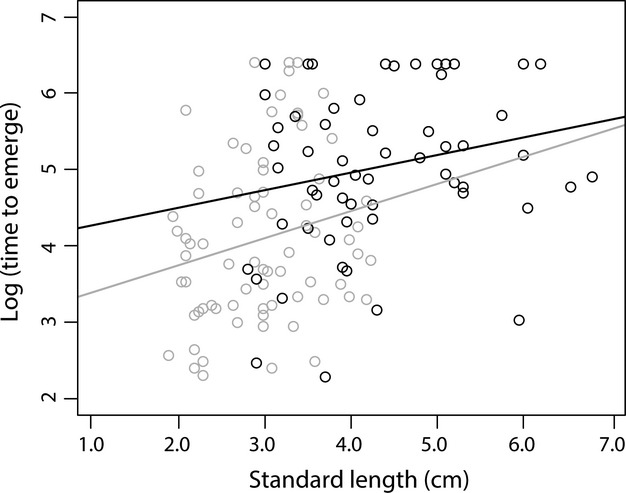
Log of time to emerge plotted as a function of standard length (cm) and line of best fit for *Brachyrhaphis roseni* (gray line and circles) and *Brachyrhaphis terrabensis* (black line and circles). There was no significant relationship between time to emerge and standard length when species were analyzed separately.

For both species, females were significantly larger than males (*B. roseni: F*_1, 73_ = 110, *P <* 0.001; *B. terrabensis*: *F*_1, 57_ = 53.293, *P <* 0.001), and *B. terrabensis* was significantly larger than *B. roseni* (*F*_1, 132_ = 88.296, *P <* 0.001). We found evidence that both species and sex had a significant effect on time to emerge, whereas SL did not (Table 1). Within species, *B. roseni* males had shorter time to emerge scores than females (*F*_1, 72_ = 5.598, *P* = 0.02), but that the difference in time to emerge between males and females of *B. terrabensis* did not significantly differ (*F*_1, 56_ = 3.331, *P* = 0.073), although the trend was for males to emerge sooner than females (Fig. 1). There was a significant positive relationship between SL and time to emerge (*F*_1, 132_ = 26.02, *P <* 0.001) when we included both species and sexes, indicating that across both species and sexes, “boldness” decreased as SL increased. This pattern was likely driven by large difference in SL between species and overall differences in the time to emerge (i.e., size effect was confounded with species). However, when we conducted regression analyses within species (both males and females combined), we found that the relationship between time to emerge and SL for *B. roseni* (*F*_1, 73_ = 3.393 *P* = 0.0696) and *B. terrabensis* (*F*_1, 57_ = 3.039, *P* = 0.0867) were nonsignificant, although the relationship between time to emerge and size had a positive trend (Fig. 2). Regression analyses within sexes for each species revealed no significant relationship between “boldness” and SL (*B. roseni* males: *F*_1, 35_ = 2.291, *P* = 0.139; *B. roseni* females: *F*_1, 35_ = 1.122, *P* = 0.297; *B. terrabensis* males: *F*_1, 32_ = 2.932, *P* = 0.097; *B. terrabensis* females: *F*_1, 23_ = 0.263, *P* = 0.613).

**Table 1:** Results from the ANCOVA, with species and sex as main effects, and SL as a covariate. Significant *P*-values are shown in bold. Effect size is included as partial *η*^*2*^

ANCOVA						
Effect	df	Sum Sq	Mean Sq	*F*-value	*P*	Partial *η*^*2*^
Species	1	29.494	29.494	27.658	**<0.001**	0.179
Sex	1	9.777	9.777	9.168	**0.003**	0.068
SL	1	0.281	0.281	0.266	0.609	0.002
Species × Sex	1	0.132	0.132	0.124	0.726	<0.001
Species × SL	1	0.103	0.103	0.097	0.756	<0.001
Species × Sex × SL	2	6.016	3.008	2.821	0.063	0.043

## Discussion

The primary objective of our study was to test for divergence in boldness traits between sister species of *Brachyrhaphis* fishes from different predation environments. Our results provide strong evidence that boldness, measured as the latency to emerge from a shelter into a novel environment, is strongly correlated with differences in predation environment (i.e., the presence of predators and corresponding environmental differences) in *Brachyrhaphis roseni* and *B. terrabensis*. As we predicted, individuals from predator-rich environments (*B. roseni*) were bolder than individuals from predator-free environments (*B. terrabensis*). These conclusions are consistent with several studies of boldness in other taxa with populations from different predation environments (Brown and Braithwaite 2004; Brown et al. 2005; Harris et al. 2010; Archard et al. 2012), including other fish species in the family Poeciliidae. Our results, along with those of other similar studies, suggest that these environmental differences are driving the observed behavioral divergence. However, further tests are necessary to determine *which* environmental differences (e.g., predation pressure, resource availability, or population density) act as the primary selective forces driving behavioral divergence, and if these behavioral traits are truly adaptive.

### Divergence in boldness traits associated with divergent environments?

*Brachyrhaphis roseni* and *B. terrabensis* occur in vastly different environments throughout their range in western Panama and southeastern Costa Rica. *Brachyrhaphis terrabensis* occupies high-elevation streams that are characterized by higher stream flow, cooler water, and very low levels of fish diversity with a lack of predators (*B. terrabensis* is often the only species present). In contrast, *Brachyrhaphis roseni* occupies low-elevation streams, characterized by low stream flow, warm water, and a diverse fish community that includes numerous potential competitors (e.g., other poeciliids and several species of Characidae, Rivulidae, among others) and several types of large piscine predators (e.g., *Hoplias microlepis* and *Gobiomorus dormitor*). We predicted that these environmental differences would be correlated with dramatically different behavioral traits, with *B. roseni* being bolder than *B. terrabensis*. Our results support this prediction.

*Brachyrhaphis roseni* emerged from a shelter into a novel environment more quickly than *B. terrabensis*. Latency to emerge from a shelter into a novel environment is a common measure of boldness and assumes that individuals that emerge from a “safe” enclosed environment into a “risky” open environment more quickly are more bold (Toms et al. 2010). According to this measure, our results provide strong evidence that *B. roseni* is prone to engage in more risky (i.e., prone to danger) behavior than *B. terrabensis*. Numerous studies have found similar patterns of behavioral divergence in populations or species from divergent predation environments. For example, studies of a congener *Brachyrhaphis episcopi* found evidence that populations from low-elevation streams with predators are more active and prone to explore (Archard and Braithwaite 2011a), and also emerge more quickly from a shelter into both a novel (Brown and Braithwaite 2004) and natural environment (Brown et al. 2005) than populations that do not co-occur with predators. Other studies have found direct evidence for potential benefits of increased activity and boldness in environments containing predators. For example, bold Trinidadian guppies (*Poecilia reticulata*) were better able to identify and escape from a potential cichlid predator and also attract more female mates (Godin and Dugatkin 1996). Furthermore, bolder three-spined stickleback fish ate a significantly greater number of prey than less bold fish, which seems to reflect their ability to perceive a trade-off between risk in a new environment and a food reward (Ioannou et al. 2008). Thus, bold behavior could benefit individuals that occur in risky, complex environments by increasing predator detection and attack survival, mating success, and the acquisition of resources. Further studies in *Brachyrhaphis* would benefit from evaluating the potential risk-benefit trade-offs of boldness.

### Sex matters: intersexual differences in boldness

Our second objective in this study was to test for differences in boldness traits between the sexes to see if males and females differed, with the prediction that males would be bolder than females due to differences in life-history strategies. Our results provide evidence that males are bolder than females in that they emerged from a shelter into a novel environment sooner than females (Fig. 1). *Brachyrhaphis roseni* males emerged significantly sooner than females, and *B. terrabensis* males tended to emerge sooner (although the difference was nonsignificant, potentially due to increase behavioral variance in the individuals sampled). It is possible that the decreased magnitude of divergence between male and female *B. terrabensis* relative to between sex divergence in *B. roseni* is due to differences in population density between the two species. Populations of *B. roseni* tend to be less dense than populations of *B. terrabensis* due to increased predator-induced mortality. Differences in boldness between these species appear to be correlated with differences in exploratory behavior and activity levels (Ingley et al. 2014b). Thus, selection might favor increased boldness, activity, and exploration levels in male *B. roseni* relative to male *B. terrabensis* to compensate for lower population densities common in high-predation environments.

General differences in boldness between males and females could stem from divergent mating systems (Harris et al. 2010). Females spend little time looking for mates because potential mating partners often surround them. Furthermore, female poeciliids have the ability to store sperm, thus allowing them to produce broods long after copulation even in the absence of males. This reproductive strategy favors behaviors that will increase longevity (e.g., risk avoidance) because fecundity is positively correlated with body size and females continue to grow throughout their lives (Reznick et al. 1996). Thus, large, long-lived females tend to have higher fecundity than small, short-lived females. In contrast, males do not have indeterminate growth and maximize their reproductive fitness by mating throughout their lifetime, thus favoring behaviors that increase encounters with potential mates, consequently increasing mating opportunities. Therefore, it is not surprising that males were bolder than females in our study. Our results align with several previous studies, in which males were more bold than females, as measured either by time spent out of a safe refuge (King et al. 2013) or time to emerge into a novel environment (Harris et al. 2010), as measured in our study.

### Does size matter?

Our third objective in this study was to test for significant associations between size and boldness across our entire data set, within species, and within sexes within species. Several lines of evidence suggest that body size and metabolism should influence boldness traits (Martin and Lopez 2003; Careau et al. 2008). For example, small fish typically have higher metabolic rates than larger fish, which can result in higher energy demands. This increased demand in energy is thought to cause them to be less averse to risk because they need to quickly resume foraging activity (Krause et al. 1998; Dowling and Godin 2002; Brown and Braithwaite 2004; Brown et al. 2005).

Prior to controlling for the effects of sex and species, we found a strong positive relationship between size and boldness. However, when we controlled for species (species differed significantly in size), and then sex and species, we found no evidence for a relationship between size and boldness. Hence, metabolic demand does not appear to explain behavior in this case. These results are thus consistent with past studies of several fish groups [rainbow trout (Johnsson 1993), killifish (Fraser et al. 2001), guppies (Harris et al. 2010) and sticklebacks (Bell and Sih 2007)], which also found no correlation between size and boldness. Surprisingly, populations of the congener *B. episcopi* from different predation environments do show a strong correlation between size and boldness (Brown and Braithwaite 2004; Brown et al. 2005). It is possible that *B. roseni* and *B. terrabensis* do not show such a relationship because size and behavior differences have become more fixed post-speciation, whereas *B. episcopi* populations have only recently diverged. In both cases, divergent predation environments are highly correlated with divergent body sizes (Jennions and Telford 2002, Belk et al. in review), although differences between *B. roseni* and *B. terrabensis* are more extreme than populations within *B. episcopi*. Further work evaluating the relationship between size and boldness in other species of *Brachyrhaphis* that have populations that occur in divergent predation environments (e.g., *B. rhabdophora*) could help shed light on this issue.

## Conclusions

Our study provides further evidence that high-predation environments, which also tend to be more complex, favor the evolution of more bold behaviors. *Brachyrhaphis roseni,* which occurs in high-predation environments, emerged from a shelter into a novel environment more quickly than *B. terrabensis,* which occurs in predator-free environments. Furthermore, we found evidence that sex was an important driver of behavioral divergence, such that males of both species tended to be bolder than females. These intersexual differences in boldness possibly reflect sex-specific differences in life-history strategies, such that males tend to be more active and bold than females in order to increase mating opportunities, while females adopt a less-risky behavioral phenotype in order to increase longevity and thus fecundity. Finally, we found no evidence that size affects boldness once we controlled for sex- and species-specific size differences, suggesting that interindividual metabolic differences are not the primary driver of behavioral divergence in this system. Our study highlights the role that environment can play in the origin of behavioral differences and provides an example of how behavioral differences can persist once speciation is complete.
